# Pathological Complete Response after Surgery following Chemotherapy with Immune Checkpoint Inhibitors for Gastric Cancer with Liver Metastases: Two Case Reports

**DOI:** 10.70352/scrj.cr.25-0649

**Published:** 2026-03-27

**Authors:** Kazuki Nishino, Michitaka Honda, Hirohito Kakinuma, Ryuya Yamamoto, Soshi Hori, Kaho Koide, Masamichi Suzuki, Noriyuki Uesugi, Tamotsu Sugai, Nobuyasu Suzuki

**Affiliations:** 1 Department of Minimally Invasive Surgical and Medical Oncology, Fukushima Medical University, Fukushima, Fukushima, Japan; 2 Department of Surgery, Southern TOHOKU Research Institute for Neuroscience, Southern TOHOKU General Hospital, Koriyama, Fukushima, Japan; 3 Department of Pathology, Southern TOHOKU Research Institute for Neuroscience, Southern TOHOKU General Hospital, Koriyama, Fukushima, Japan

**Keywords:** gastric cancer, liver metastases, nivolumab, pathological complete response

## Abstract

**INTRODUCTION:**

In recent years, advances in pharmacotherapy, including the introduction of immune checkpoint inhibitors (ICIs), have affected treatment strategies for advanced gastric cancer. Surgical intervention after systemic therapy has the potential to improve the prognosis of patients with advanced gastric cancer and liver metastases. Herein, we report two cases in which gastrectomy and hepatectomy were performed after pharmacotherapy with ICIs, resulting in a pathological complete response.

**CASE PRESENTATION:**

Case 1 was a 75-year-old man diagnosed with gastric cancer causing stenosis from the antrum to the duodenal bulb. Contrast-enhanced CT revealed a 3.2 cm liver metastasis with a tumor thrombus in the right portal and posterior sectoral branches. The patient was diagnosed with cT4aN+M1(HEP) Stage IVB gastric cancer and received four courses of S-1 plus oxaliplatin (SOX) and nivolumab. Marked tumor shrinkage led to surgery, including distal gastrectomy and posterior segmentectomy. Pathological examination revealed no residual tumor in the primary or liver lesions, with a histological grade 3 response. Postoperatively, S-1 and nivolumab were initiated, and the patient remained recurrence-free for 5 months. Case 2 was a 77-year-old man who was diagnosed with gastric cancer during screening. Contrast-enhanced CT revealed a 2.2 cm liver metastasis in segment 8. A diagnosis of cT4aN+M1(HEP) stage IVB disease was made, and he received four courses of SOX and nivolumab preoperatively. After tumor shrinkage, total gastrectomy and S8 partial hepatectomy were performed. Pathological examination revealed no residual tumor in the primary or liver lesions, with a histological grade 3 response. Postoperatively, S-1 and nivolumab were initiated, and the patient remained recurrence-free for 6 months.

**CONCLUSIONS:**

Curative resection following pharmacotherapy with ICIs may represent a potential treatment strategy for gastric cancer with liver metastases. Further case reports are required to evaluate the safety and long-term outcomes of surgery following systemic therapy.

## Abbreviations

CLDN18.2
: claudin-18 isoform 2
CPS
: combined positive score
dMMR
: deficient mismatch repair
HER2
: human epidermal growth factor receptor 2
ICI
: immune checkpoint inhibitor
NAC
: neoadjuvant chemotherapy
NLR
: neutrophil to lymphocyte ratio
pCR
: pathological complete response
PD-L1
: programmed death-ligand 1
pMMR
: proficient mismatch repair
SOX
: S-1 plus oxaliplatin

## INTRODUCTION

Gastric cancer is currently the fifth most commonly diagnosed cancer and the third leading cause of cancer-related death worldwide.^1)^ Systemic chemotherapy is the primary treatment for stage IVB advanced gastric cancer.^2,3)^ In recent years, the development of pharmacotherapies, including ICIs, has been associated with improved survival in patients with metastatic or unresectable gastric cancer.^4,5)^ The clinical significance of surgical intervention for oligometastatic disease remains unclear. Furthermore, many uncertainties, including whether ICIs are effective in the treatment of hepatic metastases, still remain.

Although the number of cases with R0 resection is increasing, reports demonstrating a pCR in both the primary lesion and liver metastases remain limited. Here, we report two cases of advanced gastric cancer with liver metastases in which gastrectomy and hepatic resection following systemic therapy, including ICIs, resulted in pCR. Such pCR involving both the primary gastric cancer and liver metastases have been reported only in limited case reports, highlighting the uncommon nature of these findings.

## CASE PRESENTATION

### Case 1

**Fig. 1:**
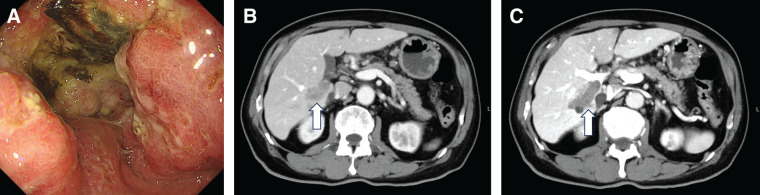
Pretreatment imaging findings. (**A**) Upper gastrointestinal endoscopy revealed a Borrmann type 3 gastric cancer with stenosis extending from the antrum to the duodenal bulb. (**B**) Contrast-enhanced CT showed a 3.2-cm liver metastasis in segment 6 (S6) (white arrow). (**C**) Contrast-enhanced CT showed a tumor thrombus in the right portal vein and posterior sectoral branch (white arrow).

A 75-year-old man, who presented with nausea, underwent an upper gastrointestinal endoscopy at a referral hospital, which revealed Borrmann type 3 gastric cancer with stenosis invading the antrum to the duodenal bulb and diagnosed histologically as poorly differentiated adenocarcinoma (**Fig. 1A**). Contrast-enhanced CT demonstrated enlarged regional lymph nodes and a 3.2-cm metastasis in segment 6; this was accompanied by a tumor thrombus in the right portal vein and posterior sectoral branches (**Fig. 1B**, **1C**). Based on these findings, the patient was diagnosed with gastric cancer cT4aN+M1(HEP), cstage IVB. Owing to obstructive symptoms, diagnostic laparoscopy and gastrojejunostomy were performed. Intraoperative findings revealed no peritoneal dissemination, and cytology of peritoneal lavage fluid was classified as class II. Immunohistochemistry of a biopsy specimen showed HER2-negative, CLDN18.2-negative, PD-L1 CPS ≥ 1, and <10 using the PD-L1 22C3 antibody, and pMMR.

**Fig. 2:**
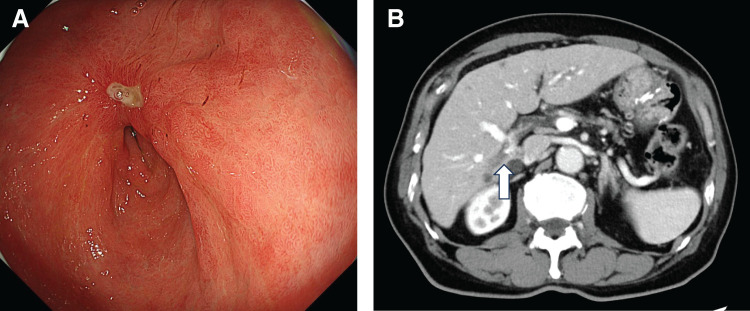
Post-chemotherapy imaging findings. (**A**) Upper gastrointestinal endoscopy showed marked regression of the gastric lesion. (**B**) Contrast-enhanced CT revealed that the liver metastasis in S6 had decreased in size to 2.1 cm (white arrow).

The patient received four courses of NAC as first-line treatment with S-1 (120 mg/body on days 1–14, every 3 weeks), oxaliplatin (170 mg/body on day 1, every 3 weeks), and nivolumab (360 mg/body on day 1, every 3 weeks), without adverse events. Post-treatment endoscopy showed primary lesion regression, and contrast-enhanced CT revealed shrinkage of lymph nodes and liver metastases (liver metastasis reduced to 2.1 cm) (**Fig. 2**). PET-CT showed uptake only at the primary site, with no uptake in the lymph nodes or liver metastasis. The clinical diagnosis was revised to ycT4aN+M1(HEP), ycstage IVB, and surgery was planned.

The patient underwent open distal gastrectomy with D2 lymphadenectomy and posterior segmentectomy of the liver. Intraoperatively, fibrosis was noted at the duodenal invasion site due to chemotherapy; however, no evidence of pancreatic invasion was found, allowing for safe dissection. Intraoperative ultrasonography failed to detect the liver metastasis or tumor thrombus; however, owing to the absence of blood flow in the posterior sectoral branch, a posterior segmentectomy was performed. Operative time was 347 min, with 450 mL of blood loss. On POD 1, a duodenal stump leak was identified. Drainage catheters were placed under general anesthesia, and the patient was successfully treated with drainage and antibiotics. The patient was discharged on POD 29.

**Fig. 3:**
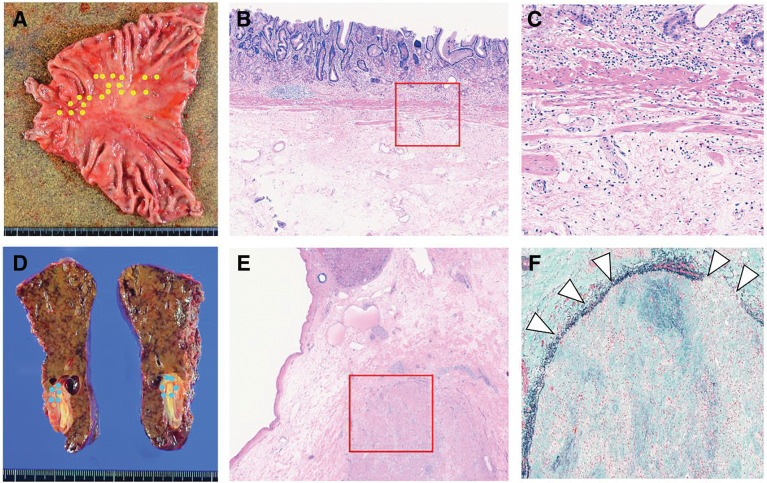
Surgical specimens and histopathological findings. (**A**) Resected stomach specimen showing fibrotic changes at the tumor site (yellow dotted line). (**B**) Histopathological image of the stomach (hematoxylin and eosin [H&E] stain, ×25) showing fibrosis from the submucosa to the muscularis propria. (**C**) Histopathological image of the stomach (H&E, ×100) showing clusters of foamy macrophages and fibrotic scar formation, with no viable tumor cells detected; this represents a higher magnification view of the red boxed area in (**B**). (**D**) Resected liver specimen showing partial fibrosis at the tumor site (blue dotted line). (**E**) Histopathological image of the liver (H&E, ×12.5) showing fibrotic tissue in the liver parenchyma. (**F**) Histopathological image of the liver (Elastica Masson [EM] stain, ×50) showing fibrotic tissue within the portal vein, with no viable tumor cells (white arrowhead); this represents a higher magnification view of the red boxed area in (**E**).

Pathological examination revealed fibrotic scar tissue in the primary and liver metastatic lesions, with no viable tumor cells detected. Fibrotic tissue was also observed within the portal vein, and the histological response was assessed as grade 3 (**Fig. 3**). Postoperatively as part of a consistent treatment of strategy for stageIV gastric cancer, S-1(100 mg/body on days 1–14, every 3 weeks for 14 days) and nivolumab (360 mg/kg body on day 1, every 3 weeks) was continued. Oxaliplatin was discontinued due to the development of peripheral neuropathy. The patient remained recurrence-free at 5 months postoperatively.

### Case 2

**Fig. 4:**
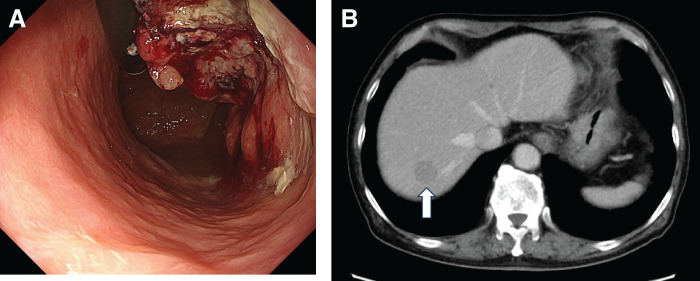
Pretreatment imaging findings. (**A**) Upper gastrointestinal endoscopy revealed a Borrmann type 2 gastric cancer on the anterior wall of the upper to middle third of the stomach. (**B**) Contrast-enhanced CT demonstrated a 2.2-cm liver metastasis in segment 8 (S8) (white arrow).

A 77-year-old man was diagnosed with gastric cancer after an upper gastrointestinal barium study. Subsequent endoscopy revealed a Borrmann type 2 tumor located on the anterior wall of the upper to middle third of the stomach, and histologically confirmed as moderately differentiated tubular adenocarcinoma (**Fig. 4A**). Contrast-enhanced CT demonstrated enlarged lymph nodes along the lesser curvature and a 2.2-cm liver metastasis in segment 8 (**Fig. 4B**). Based on these findings, the patient was diagnosed with stage IVB gastric cancer, cT4aN+M1 (HEP). Immunohistochemistry of the biopsy specimen showed HER2-negative, CLDN18.2-negative, PD-L1 CPS ≥5 using the PD-L1 28-8 antibody, and pMMR.

**Fig. 5:**
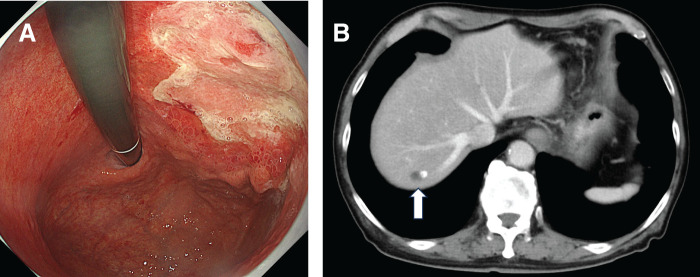
Post-chemotherapy imaging findings. (**A**) Upper gastrointestinal endoscopy showed marked regression of the gastric lesion. (**B**) Contrast-enhanced CT revealed that the liver metastasis in segment 8 decreased in size to 1.0 cm (white arrow).

Four courses of S-1 (120 mg/body on days 1–14 every 3 weeks), oxaliplatin (160 mg/body on day 1, every 3 weeks), and nivolumab (360 mg/body on day 1, every 3 weeks) were administered as NAC, with no adverse events observed. Post-treatment endoscopy showed tumor regression, and contrast-enhanced CT revealed shrinkage of the lymph nodes and liver metastases (the liver lesion decreased to 1.0 cm) (**Fig. 5**). The clinical diagnosis was revised to ycT4aN+M1(HEP), ycstage IVB, and surgery was planned.

The patient underwent open total gastrectomy with D2 lymphadenectomy and Roux-en-Y reconstruction, along with partial hepatectomy of segment 8. Operative time was 370 min with 365 mL of blood loss. On POD 7, the patient developed sepsis due to prostatitis, which improved with antibiotic therapy, and he was discharged on POD 18.

**Fig. 6:**
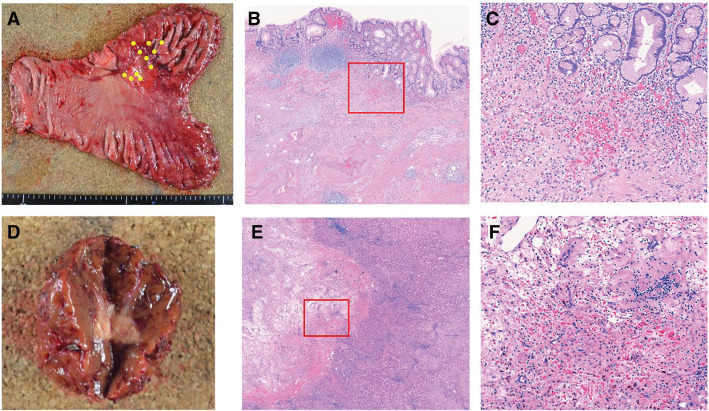
Surgical specimens and histopathological findings. (**A**) Resected stomach specimen showing fibrotic changes at the tumor site (yellow dotted line). (**B**) Histopathological image of the stomach (hematoxylin and eosin [H&E], ×25) showing fibrosis from the submucosa to the muscularis propria. (**C**) Histopathological image of the stomach (H&E, ×100) showing clusters of foamy macrophages and fibrotic scar formation, with no viable tumor cells dectected; this represents a higher magnification view of the red boxed area in (**B**). (**D**) Resected liver specimen showing partial necrosis of the tumor macroscopically. (**E**) Histopathological image of the liver (H&E, ×12.5) showing partial fibrosis of the tumor site. (**F**) Histopathological image of the liver (H&E, ×100) showing diffuse infiltration of foamy macrophages, with no viable tumor cells; this represents a higher magnification view of the red boxed area in (**E**).

Pathological examination revealed clusters of foamy macrophages and fibrotic scar formation in the primary and liver lesions, with no viable tumor cells. Histological therapeutic effect was assessed as grade 3 (**Fig. 6**). Postoperative adjuvant chemotherapy with S-1 (120 mg/body on days 1–14 every 3 weeks) and nivolumab (360 mg/body on day 1, every 3 weeks) was continued as part of a consistent treatment strategy for stageIV gastric cancer. Oxaliplatin was discontinued due to the development of peripheral neuropathy. The patient remained recurrence-free at 6 months postoperatively.

## DISCUSSION

Here, we report two cases of advanced gastric cancer with solitary hepatic metastasis that were treated with resection following induction chemotherapy with ICIs. Both patients tested negative for HER2 and CLDN18.2. According to the Japanese Gastric Cancer Treatment Guidelines, the standard first-line regimen in such cases is a combination of fluoropyrimidine, platinum-based chemotherapy, and ICIs.^6)^ After four courses of therapy, radiological assessment demonstrated sufficient tumor response, and PET imaging showed no uptake in the liver metastases, indicating good disease control. Based on these findings, R0 resection was considered feasible, and surgery was performed at that time. The treatment strategy for stage IV gastric cancer, including the indication for surgery, is determined based on the Gastric Cancer Treatment Guidelines and the Yoshida classification proposed in the CONVO-GC-1 study.^6,7)^ At our institution, the criteria for determining surgical eligibility after neoadjuvant chemotherapy for gastric cancer cases with oligo-metastases are as follows: maintaining a good response for at least 3 months and showing no new lesions on whole-body imaging, including PET-CT. Given the limited evidence and lack of expert consensus for this treatment, we require thorough patient understanding and informed consent, as well as approval from our institution’s multidisciplinary cancer board. Pathological examination revealed pCR in the primary gastric lesions and hepatic metastases. These cases highlight therapeutic considerations for oligometastatic disease and provide insights into the potential efficacy of ICIs in treating hepatic metastases from gastric cancer.

Prospective interventional studies that evaluated the role of surgical resection in oligometastatic gastric cancer treatment demonstrated that patients with limited metastases involving only one organ, such as the para-aortic lymph nodes, liver, or lungs, show better prognoses than those with multiple metastatic sites.^8)^ However, these studies were non-randomized and included a small number of resectable liver metastases; therefore, an optimal treatment strategy for hepatic oligo-metastasis has not yet been established. Retrospective studies have reported that hepatic resection for solitary liver metastases from gastric cancer is associated with prolonged survival.^9–13)^ Fujitani et al. examined outcomes of hepatectomy following neoadjuvant chemotherapy and reported that patients with solitary liver metastases who achieved R0 resection had a median overall survival of 39.8 months.^14)^ The CONVO-GC-1 trial also investigated outcomes of hepatic resection in stage IV gastric cancer, suggesting that patients with R0 resection and a pathological response of grade ≥2 achieved favorable prognoses.^7)^ In particular, in cases with a single hepatic metastasis of <5 cm, R0 resection was achieved in 85.2% of patients, with a reported median survival of 95.2 months. Collectively, these findings support the rationale for combining chemotherapy with surgical resection in selected patients with solitary liver metastases. With the increasing use of ICIs, further improvements in long-term outcomes are anticipated, and surgical treatment may play an increasingly important role in the therapeutic strategy for oligometastatic gastric cancer.

However, retrospective analyses comparing the outcomes of hepatectomy and chemotherapy in patients with 2 to 3 hepatic metastases have not demonstrated a survival benefit with resection.^15)^ Moreover, multiple liver metastases remain a poor prognostic factor, and the role of conversion surgery in such cases has not been established.^16)^ A clinical trial addressing this issue is currently underway by the Japan Clinical Oncology Group (JCOG).

Reports on the tumor-shrinking effects of PD-L1 blockade in hepatic metastases from gastric cancer remain limited. A recent study indicated that therapeutic efficacy does not significantly differ between patients with and without hepatic metastases; however, no case series has demonstrated histopathological responses in resected liver lesions.^17)^

A PubMed search identified seven cases in which gastrectomy and hepatectomy were performed simultaneously after chemotherapy with ICIs.^18–22)^ Among these, 3 cases, similar to our own, achieved pCR in the primary gastric tumor and metastatic lesion (**Table 1**). Reported treatment regimens included SOX plus nivolumab, and combinations with molecular-targeted agents or invariant natural killer T cell-based immunotherapy. The therapeutic efficacy of ICIs has been reported to be higher in patients with a PD-L1 CPS ≥10 and in those with dMMR.^5,23,24)^ In a previous report summarizing patients with metastatic gastric cancer and gastroesophageal junction cancer who achieved a clinical complete response (CR) following systemic chemotherapy, the proportions of HER2 positivity (23.5%), CLDN18.2 positivity (16.0%), CPS ≥10 (30.0%), and dMMR (23.3%) were relatively high.^25)^

**Table 1:** Summary of cases undergoing simultaneous gastrectomy and hepatectomy following chemotherapy with ICIs

Case	References	Years	Age	Sex	MMR	CPS	Regimen (course)	Operation	HR Grade (gastric/liver)	Postoperative chemotherapy	RFS (months)	Outcome
1^18)^	Li	2022	60	M	–	1	SOX,Cam, iNKT cells(2)	TG, PH	3/3	None	11	Alive
2^19)^	Ueno	2023	82	M	–	≥5	SOX, Nivo(2)	DG, PH	1b/3	S-1	6	Alive
3^20)^	Katsumata	2024	54	M	–	≥5	SOX, Nivo(4)	TG, PH	3/3	S-1	18	Alive
4^21)^	Kawai	2024	68	M	pMMR	≥5	SOX, Nivo(13)	DG, PH	1a/3	S-1, DTX	7	Alive
							Nivo(3)					
5^21)^	Kawai	2024	72	M	–	0	SOX(23)	PG, PH	1a/3	None	7	Alive
							Ram, PTX(1)					
							Nivo					
6^21)^	Kawai	2024	51	F	–	≥5	SOX, Nivo(10)	DG, PH	1b/3	S-1, DTX	6	Alive
							Nivo(5)					
7^22)^	Ogura	2024	70	M	–	None	SOX(7)	PG, PH	3/3	Nivo	8	Alive
							Ram(8)					
							Nivo(6)					
8	Our case 1	2025	75	M	pMMR	≥1	SOX, Nivo(4)	DG, PS	3/3	S-1, Nivo	5	Alive
						<10						
9	Our case 2	2025	77	M	pMMR	≥5	SOX, Nivo(4)	TG, PH	3/3	S-1, Nivo	6	Alive

Cam, camrelizumab; CPS, combined positive score; DG, distal gastrectomy; DTX, docetaxel; F, female; HR, histological response; ICI, immune checkpoint inhibitor; iNKT, invariant natural killer T; M; male; MMR, mismatch repair; Nivo, Nivolumab; PG, proximal gastrectomy; PH, partial hepatectomy; PS, posterior segmentectomy; PTX, paclitaxel; Ram, ramucirumab; RFS, relapse-free survival from conversion surgery; SOX, S-1+oxaliplatin; TG, total gastrectomy

By contrast, in our cases, Case 1 exhibited pMMR with a low CPS, and Case 2 showed CPS ≥5 but retained pMMR, both of which are generally considered clinical features associated with a lower likelihood of response to ICIs. In addition, an association between the therapeutic response to ICIs and the NLR has been reported. In patients with gastric cancer treated with ICIs, a high NLR has been shown to be associated with poorer overall survival, suggesting that NLR may serve as a potential predictive biomarker for the efficacy of ICI therapy.^26)^ In our cases, the baseline NLR prior to nivolumab administration was 2.5 in Case 1 and 4.7 in Case 2. Although Case 1 showed a relatively low NLR, Case 2 had a moderately elevated value. These findings suggest that NLR alone may not fully predict clinical response to ICIs. Treatment duration, timing of surgery, and postoperative management varied across reports, and no consensus has been established. Postoperative strategies included continuation of chemotherapy, molecular-targeted therapy, ICIs, and observation without further therapy in cases achieving pCR. In the ATTRACTION-5 trial, postoperative adjuvant nivolumab combined with chemotherapy did not show a significant improvement in disease-free or overall survival compared with chemotherapy alone, indicating that its benefit in the postoperative adjuvant setting remains unproven.^27)^ In our cases, postoperatively as part of the treatment strategy for stage IV gastric cancer, SOX plus nivolumab was continued. Oxaliplatin was discontinued because of the development of peripheral neuropathy. It is likely that treatment decisions were individualized based on the severity of chemotherapy-related adverse events, surgery-related morbidity specific to gastrectomy, and patient condition. In existing reports, no recurrence has been documented during follow-up; however, these regimens are relatively novel, and the observation periods remain short, necessitating further accumulation of long-term data.

A pCR following preoperative chemotherapy is associated with favorable prognosis. In recent years, multiple positive clinical trials have reported gastric cancer pharmacotherapy, demonstrating that the addition of ICIs to cytotoxic chemotherapy enhances tumor shrinkage, thereby increasing interest in conversion surgery.^5,28)^ Among patients with stage IVB disease, those with oligometastatic status represent a subgroup most likely to benefit from surgical intervention, underscoring the value of accumulating case reports. Future challenges include identification of clinical predictors of pCR, determination of optimal resection timing, clarification of long-term prognostic benefits, and establishment of standardized postoperative adjuvant therapy.

## CONCLUSIONS

We encountered two cases of gastric cancer with liver metastases that achieved pCR following surgery after chemotherapy with ICIs. Further accumulation of such cases is needed to evaluate long-term outcomes.

## DECLARATIONS

### Availability of data and materials

All data generated during this study are included in this published article.
